# Survival rate in patients with hepatocellular carcinoma: a retrospective analysis of 389 patients

**DOI:** 10.1038/sj.bjc.6602590

**Published:** 2005-05-03

**Authors:** T F Greten, F Papendorf, J S Bleck, T Kirchhoff, T Wohlberedt, S Kubicka, J Klempnauer, M Galanski, M P Manns

**Affiliations:** 1 Department of Gastroenterology, Hepatology and Endocrinology, Medizinische Hochschule, Carl Neuberg Str 1, Hannover, 30625 Germany; 2 Department of Diagnostic Radiology, Medizinische Hochschule, Hannover, Germany; 3 Department of Visceral and Transplantation Surgery, Medizinische Hochschule, Hannover, Germany; 4 Cancer Center, Medizinische Hochschule, Hannover, Germany

**Keywords:** chemoembolisation, liver transplantation, cancer, PEI

## Abstract

Hepatocellular carcinoma (HCC) is the fifth most common cancer worldwide. However, treatment options are limited and often inefficient. The aim of this study was to determine current survival rates for patients diagnosed with HCC and to identify prognostic factors, which will help in choosing optimal therapies for individual patients. A retrospective analysis of medical records was performed on 389 patients who were identified through the central tumour registry at our institution from 1998 to 2003. Clinical parameters, treatments received and survival curves from time of diagnosis were analysed. Overall median survival was 11 months. Liver cirrhosis was diagnosed in 80.5% of all patients. A total of 170 patients received transarterial chemoembolisation (TACE) and/or percutaneous ethanol injections (PEI) with a median survival rate of 16 months for patients receiving TACE, 11 months for patients receiving PEI and 24 months for patients receiving TACE followed by PEI. Independent negative prognostic parameters for survival were the presence of portal vein thrombosis, advanced liver cirrhosis (Child–Pugh score B or C) and a score of >2. This study will help to estimate survival rates for patients with HCC according to their clinical status at diagnosis and the treatments received.

Hepatocellular carcinoma (HCC) is the fifth most common cancer worldwide, and the fourth most common cause of cancer-related death (Parkin *et al*, 2001; Bosch *et al*, 2004). Although HCC occurs more frequently in Southeast Asia and Africa, its incidence in Western countries has almost doubled in the past 20 years (El-Serag, 2004) due to an increase of hepatitis C virus and alcohol-induced liver cirrhosis (Morgan *et al*, 2004).

Curative resection of HCC is possible (Hoofnagle, 2004), however the success of this approach is limited because of the high rate of tumour recurrence or the development of new tumours in the cirrhotic liver (Llovet *et al*, 2004). Liver transplantation has become a frequently used alternative, but it is clearly not possible for all patients and a significant number of HCC reoccur in the transplanted liver (Schlitt *et al*, 1999).

Local ablative therapies are increasingly being used to treat HCC, either as definitive therapy or as an intermediate step in patients awaiting liver transplantation. There is no standard treatment for unresectable HCC, but transarterial chemoembolization (TACE) has been shown to increase survival in a randomized controlled trial (Llovet *et al*, 2002). Percutaneous ethanol injection (PEI) has also proven to be relatively easy to perform and is inexpensive (Lencioni *et al*, 2004). Retrospective data from one Japanese centre suggests that percutaneous tumour ablation methods can become as efficient as surgical procedures (Omata *et al*, 2004).

In contrast to other cancers, the prognosis of patients with HCC is not solely related to tumour stage. Cirrhosis underlies the neoplasm in most cases and has major impact on the prognosis of patients with HCC. Accordingly, different prognostic systems assessing liver function and tumour stage have been developed such as the Okuda staging (Okuda *et al*, 1985), CLIP score (2000) and the BCLC (Llovet *et al*, 2004). Okuda staging is based on the size of the tumour, presence of ascites, serum albumin and bilirubin levels. CLIP score is based on Child–Pugh stage, tumour morphology, the presence of portal vein thrombosis and the AFP level, while the BCLC staging is similar to the Okuda staging system but includes the presence of vascular invasion if present.

Hepatocellular carcinoma is a major health problem around the world. However, the biology of the disease varies between different areas. In Asia, HBV infection is the major risk factor for HCC, whereas HCV infection and alcohol use are more frequently the cause for liver cirrhosis and HCC in the Western world (Wang *et al*, 2002). Additionally, the application of treatment options and the success with which they are applied varies as well, so that it is essential to study not only responses to therapy and overall survival in patients but also the prognostic risk factors (Stuart *et al*, 1996). Until today there is only limited data available describing the outcome related to baseline patient characteristics among all patients with HCC in Western countries.

Therefore, we have collected data from a large group of HCC patients, who were consecutively presented to our referral centre in Germany between January 1998 and December 2003. We have retrospectively examined the clinical baseline characteristics of 389 patients, as well as their respective forms of therapy and ultimate outcomes. Using uni- and multivariate regression analysis, we have attempted to construct both a descriptive evaluation of the individual subgroups and their survival and to identify possible clinically evident prognostic factors at the time of presentation. These data will help to find optimal treatment modalities for individual patients in the future and build a base for future clinical trials, when different therapy algorithms will be evaluated.

## PATIENTS AND METHODS

The central tumour registry at Medizinische Hochschule Hannover has collected data from all patients, who were presented with HCC at the Department of Gastroenterology, Hepatology and Endocrinology since 1997. We have examined the medical records of all patients who were presented at our Department between January 1998 and December 2004 to confirm the diagnosis and relevant clinical parameters. Hepatocellular carcinoma was diagnosed according to EASL guidelines (Bruix *et al*, 2001). The following parameters were examined: age, sex, clinical or pathological stage using the Okuda and CLIP score systems (Okuda *et al*, 1985; The Cancer of the Liver Italian Program, 2000). Presence or absence of cirrhosis was analysed and, if cirrhosis was present, the extent was classified according to the Child–Pugh classification (Child and Turcotte, 1964). Any risk factors for developing cirrhosis were registered including hepatitis B or C, haemochromatosis, alcohol abuse or autoimmune hepatitis. Alpha-fetoprotein levels were analysed in all patients in the central clinical biochemistry laboratory of our institution. In addition, any form of therapy undergone by the patient was recorded. Survival was determined from the time of initial diagnosis. Actuarial survival was calculated using the methods of Kaplan and Meier (1958). Baseline parameters were analysed with the log-rank test to identify potential prognostic factors. Multivariate analysis was then performed using the Cox proportional hazards regression (Cox and Oakes, 1984) including those parameters, which were significant according to the univariate test.

Survival curves were compared using the Cox–Mantel log-rank test. Univariate and multiple regression analysis of covariance among patient characteristics were performed with the aid of SSP Software.

## RESULTS

### Patient characteristics

Three hundred and eighty-nine patients were seen at the Department of Gastroenterology, Hepatology and Endocrinology between January 1998 and December 2003 with confirmed diagnosis of HCC. Patient characteristics are summarised in Table 1. The male to female ratio was 3.8 : 1 and liver cirrhosis was present in 80.5% of the cases. Viral hepatitis and alcohol were the most common cause for liver cirrhosis. The average age was 64 years old (mean 18–85 years). In all, 52% of the patients had one liver lesion, 42% had two or three lesions and the remaining patients had more than three lesions. We have used Okuda staging and CLIP score analysis to classify baseline parameters for those HCC patients, for whom detailed staging information was available. Of 319 patients, 24% were classified as Okuda I, 64% as Okuda II, and 12% as Okuda III. Twenty (8%) patients had a CLIP score of zero. Twenty-six (11%) patients had a CLIP score of one. Sixty-five (27%) patients had a CLIP score of two and 129 (54%) patients had a CLIP score above two. A total of 33 (9%) patients had metastatic disease at the time of diagnosis. Complete and partial portal vein thrombosis was found in 17 (4%) and 65 (17%) of all patients. AFP levels were elevated above 400 ng/ml in 137 (41%) patients and the elevated bilirubin levels were found in 49% of the patients.

### Treatment

**Figure 1:**
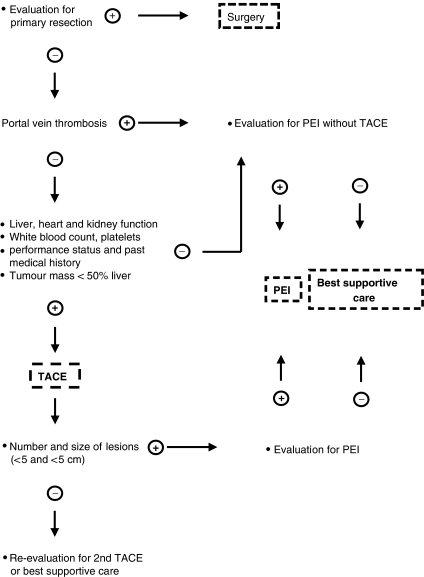
Treatment algorithm for patients with HCC.

A total of 37 patients underwent surgical therapy including liver transplantation in 12 cases. Patients, who were not eligible for primary surgery, were treated according the algorithm shown in Figure 1. If possible, all patients underwent primarily TACE followed by PEI as previously suggested by Allgaier (Allgaier *et al*, 1998). If PEI was not possible after the first TACE, this treatment was repeated if needed until the tumour progressed despite treatment. Overall, TACE was performed in 103 patients. A mixture of lipiodol, cisplatin and doxorubicin was used in most cases and no TACE-related deaths occurred. Percutaneous ethanol injection was performed in 121 patients, of which 52 patients received both, PEI and TACE. Percutaneous ethanol injection was repeated if new tumours appeared. Tables 3 and 4 show the number of PEIs performed for each group of patients. Finally, 147 patients were not eligible for either of the three treatment options (surgery, PEI and TACE) due to the extent of liver cirrhosis or tumour and were treated with sandostatin, tamoxifen, pravasin, systemic chemotherapy or best supportive care (Table 2).

### Survival

**Figure 2:**
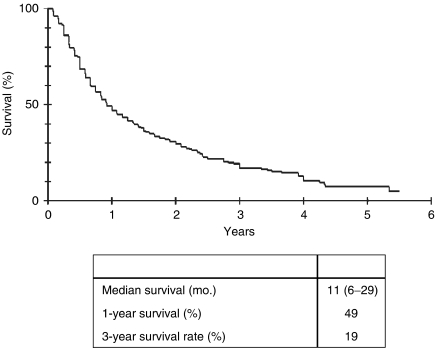
Kaplan–Meier survival curve for all 389 patients. Median survival rate was 13 months, 1- and 3-year survival rate at 53 and 22 months. Median survival is presented as month (upper – lower interquartile range).

**Figure 3:**
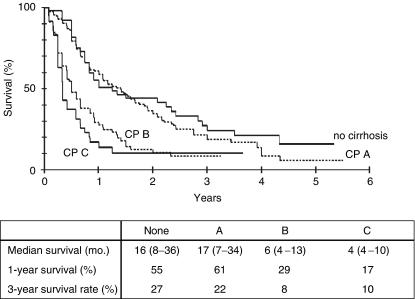
Kaplan–Meier survival curve for 355 patients depending on the presence and extent of liver cirrhosis according to Child–Pugh classification. Median survival is presented as month (upper – lower interquartile range).

Average follow-up for all HCC patients in this study was 20.4 months. Overall median survival of all 389 patients was 11 months from the date of diagnosis. The 1-year survival rate was 49%, after 3 years 19% of all patients were still alive (Figure 2). Next, patients were subdivided into different groups depending on their clinical status at the time of initial presentation at our department. If liver cirrhosis was present, the Child score system was used to categorize patients into patients with Child A, Child B and Child C cirrhosis, respectively. The 1- and 3-year survival rate differed significantly between patients without cirrhosis or Child A cirrhosis and those with Child B or C cirrhosis (Figure 3), while there was no significant difference in survival between patients with Child A cirrhosis and no cirrhosis with a median survival of 17 and 16 months, respectively. Patients with Child B cirrhosis had a median survival rate of 6 months and patients with Child C cirrhosis had the poorest median survival of 4 months.

**Figure 4:**
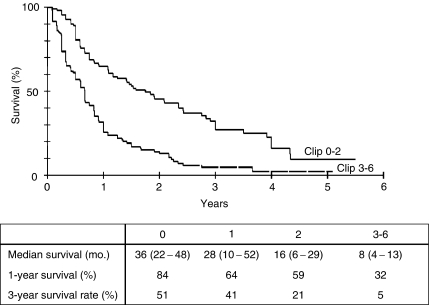
Kaplan–Meier survival curve for 240 patients depending on their initial CLIP score. Median survival is presented as month (upper – lower interquartile range).

Additionally, patient's status at initial presentation in our department was rated according to the CLIP score. As expected, patients with a CLIP score of zero points had the best median survival of 36 months (Figure 4). Patients with a CLIP score of 1–2 had a median survival of 28 and 16 months and finally patients with a CLIP score >2 points had only a median survival of 8 months.

**Figure 5:**
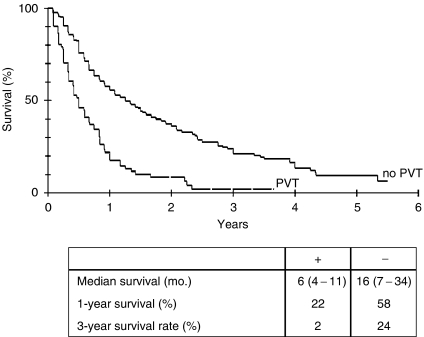
Kaplan–Meier survival curve for 341patients depending on the presence (+) or absence (−) of a portal vein thrombosis. Median survival is presented as month (upper – lower interquartile range).

We also compared the group of patients with partial and complete portal vein thrombosis, since it has been previously suggested that thrombosis of the portal vein is an independent negative predictor of ultimate survival CLIP score (2000). Indeed, we were able to confirm this observation. Patients with portal vein thrombosis had a significantly lower median survival of 6 months in contrast to patients without portal vein thrombosis, who had a median survival rate of 16 months (Figure 5).

**Figure 6:**
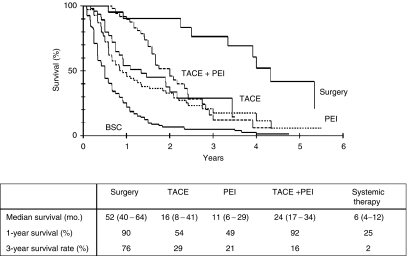
Kaplan–Meier survival curve for 288 patients depending on the therapy received. Median survival is presented as month (upper – lower interquartile range).

Finally, patient survival was analysed on the basis of the treatments that the patients received. As shown in Figure 6, there was a significant segregation in survival curves. As expected, patients who underwent surgical therapy, had the best median survival rate of 52 months, followed by patients receiving TACE and PEI (24 months), TACE (16 months) and PEI (11 months). Patients, who were not eligible for surgery, TACE or PEI had a median survival rate of 6 months.

Uni- and multivariate analysis of potential factors affecting patient survival was performed in order to identify those risk factors, which predict survival of patients. This analysis is important to identify optimal therapies for patients with HCC. Sex, age, tumour size, bilirubin, Child–Pugh score, Okuda stage, CLIP stage, the presence of one or multiple tumours, the location of the tumour in the liver were all evaluated. Only, CLIP score, Child stage and the presence of portal vein thrombosis were identified to be independent risk factors affecting patient survival as shown in Table 5 by multivariate analysis. Additionally, bilirubin, number and size of individual intrahepatic tumours were identified as independent risk factors by univariate analysis.

## DISCUSSION

We have analysed 389 patients with HCC, who were treated at our department between 1998 and 2003. In contrast to a series of other published studies (Chevret *et al*, 1999; Llovet *et al*, 1999; The Cancer of the Liver Italian Program, 2000; Villa *et al*, 2000; Rabe *et al*, 2001; Herold *et al*, 2002), this study included a high number of patients treated by TACE and PEI, which have become the most frequently used treatments in the Western World. No patient was excluded from our retrospective analysis in contrast to those studies, who investigated only the effect of surgery or local ablative therapy (Tanaka *et al*, 1998; Schlitt *et al*, 1999; O'Suilleabhain *et al*, 2003; Jaeck *et al*, 2004). Therefore, this data reflects the current situation, for unselected patients with HCC similar to a different study published 9 years ago (Stuart *et al*, 1996).

As previously reported for other non-Asian centres (Stuart *et al*, 1996; Rabe *et al*, 2001; Erhardt *et al*, 2002), our patient population differed significantly from Asian centres (Sithinamsuwan *et al*, 2000) with less HbsAg-positive patients in our study population. Moreover, the average age of our patients was 64 years old indicating that currently HCC is more frequently diagnosed in older patients as described by others (Dohmen *et al*, 2004).

In contrast to most retrospective reports, which focus on the epidemiology and risk factors contributing to development of HCC in Western countries (Kubicka *et al*, 2000; Rabe *et al*, 2001; Llovet and Beaugrand, 2003; Caselitz *et al*, 2004), our analysis was undertaken to analyse patient survival according to clinical stage and possible treatment respectively and in order to identify possible prognostic factors, which might prove to be helpful, when the best possible treatment has to be chosen for patients with HCC.

According to tumour stage, extent of liver cirrhosis and other clinical factors, different treatment options were chosen in this study as indicated in Figure 1. Therefore different treatments were not directly comparable and survival differences might be due to different stages of the disease.

The number of patients eligible for surgical resection was much smaller than the number of patients receiving TACE and PEI. In addition, the tumour stage and cirrhosis differed between these two groups leading to the observed differences in survival in contrast to studies published by others (Llovet *et al*, 2004). Similar to other reports, we saw a median survival of 24 months for the patients receiving TACE followed by PEI (Allgaier *et al*, 1998). A significant number of patients, who were not eligible for a combined treatment of TACE and PEI due to their stage of disease, were treated with TACE or PEI only. As expected these patients had lower survival rates. However, with minor differences depending on the group of patients selected and treatment modalities chosen, similar results were reported by others (Stuart *et al*, 1996; Allgaier *et al*, 1998; Bruix and Llovet, 2002; Herold *et al*, 2002; O'Suilleabhain *et al*, 2003).

Independent prognostic factors can help in assessing the individual prognosis for patients with HCC and are therefore critical for the patients. In our analysis we were able to identify CLIP score, the presence of portal vein thrombosis and extent of liver cirrhosis as independent prognostic factors and demonstrate the pivotal role of liver cirrhosis for the prognosis of patients with HCC, which has previously also been described by a number of different groups (Bruix and Llovet, 2002).

In summary, our retrospective study provides important information for the treatment of patients with HCC, while it should be emphasised that firm definite conclusions should only be drawn from prospectively randomized studies. Using a significant number of patients we have calculated survival rates for all patients with HCC regardless of therapy using different criteria such as disease state and therapy options. These data will form the basis for future randomised clinical trials evaluating new therapeutic options for the treatment of HCC.

## Figures and Tables

**Table 1:** Patient characteristics at baseline

	**Number of patients**
Total number of patients	389
Male/female	309/80
Age (mean/range)	64 (18–85)
Child (A/B/C/no cirrhosis)	155/79/35/51
Hepatitis (B/C/B+C)	57/78/7
AIH/haemachromatosis	4/17
Toxic/unknown	113/2
Nodes (1/>1)	125/259
Liver lobe (right/left/both)	135/42/208
Portal vein thrombosis (partial/complete/no)	65/17/294
OKUDA (1/2/3)	76/203/40
CLIP (0/1/2/3–6)	20/26/65/129
AFP (<400/⩾400)	196/137
Bilirubin (<17 *μ*mol/l/>17 *μ*mol/l)	173/169

**Table 2:** Initial treatments received

	**Number of patients**
Surgery^a^	25 (6.4%)
PEI	69 (17.7%)
TACE+PEI	52 (13.4%)
TACE	49 (12.6%)
Other^b^	194 (49.9%)

PEI=Percutaneous ethanol injections; TACE=transarterial chemoembolisation.

a Twelve additional patients underwent liver transplantation.

b Including best supportive care and systemic treatment with sandostatin, tamoxifen, pravasin and two patients receiving RFA and two patients receiving TACE followed by surgery.

**Table 3:** Number of PEI, which was used to treat patients with PEI only

**Number of PEIs**	**Number of patients**
1	29 (42%)
2	20 (29%)
3	7 (10%)
>3	13 (19%)

PEI=Percutaneous ethanol injections.

**Table 4:** Number of PEI, which was used to treat patients with TACE followed by PEI

**Number of PEIs**	**Number of patients**
1	17 (33%)
2	15 (29%)
3	11 (21%)
**>**3	9 (17%)

PEI=Percutaneous ethanol injections.

**Table 5:** Independent variables predictive of 5-year survival by multivariate analysis

	***P***	**Relative risk (95% CI**)
CLIP score	0.034	1.613 (1.036–2.511)
Portal vein thrombosis	<0.0001	2.443 (1.797–3.319)
Child A *vs* Child B and C	0.012	1.965 (1.160–3.329)
